# Mixed Connective Tissue Disease (MCTD): A Rare Presentation of Probable Central Nervous System (CNS) Vasculitis

**DOI:** 10.1155/crnm/1782813

**Published:** 2026-10-17

**Authors:** Arpan Mitra, Niraj Kumar Srivastava, Ankur Vivek, Nayana Bhuyan, Rukhsar Parveen, Vijaya Nath Mishra, Abhishek Pathak

**Affiliations:** ^1^ Department of Neurology Institute of Medical Sciences (IMS) Banaras Hindu University (BHU) Varanasi 221005 Uttar Pradesh India; ^2^ Department of Kaya Chikitsa, Faculty of Ayurveda Institute of Medical Sciences (IMS) Banaras Hindu University (BHU) Varanasi 221005 Uttar Pradesh India

**Keywords:** mixed connective tissue disease, mononeuritis multiplex, multiple lacunar infarcts, systemic lupus erythematosus

## Abstract

Mixed connective tissue disease (MCTD) may rarely involve the central nervous system (CNS), and CNS vasculitis can present with acute seizures, altered consciousness, and focal neurological deficits. We report a 27‐year‐old woman with a seven‐year history of arthralgia, morning stiffness, intermittent fever, and malaise who developed acute seizures, altered sensorium, and hemiparesis approximately two weeks before hospital admission. Laboratory evaluation demonstrated severe anemia, thrombocytopenia, elevated inflammatory markers, and positive anti‐U1 ribonucleoprotein (U1‐RNP) antibodies. Cerebrospinal fluid (CSF) showed lymphocyte‐predominant pleocytosis and markedly elevated protein, while brain MRI demonstrated multifocal confluent subcortical and periventricular lesions with restricted diffusion. Based on clinical, serological, CSF, and neuroimaging findings, MCTD‐associated CNS vasculitis was considered probable. The patient received broad‐spectrum antibiotics and antiseizure and antihypertensive therapy, followed by methylprednisolone pulses, oral prednisolone, and hydroxychloroquine, with significant neurological improvement at discharge. Cyclophosphamide therapy was planned; however, no subsequent follow‐up data were available. This case emphasizes the need to consider CNS involvement in patients with MCTD while recognizing the limitations of imaging specificity and incomplete long‐term follow‐up.

## 1. Introduction

Mixed connective tissue disease (MCTD) was first described as a distinct clinical entity by Sharp et al. in 1972 [1]. It is a systemic autoimmune rheumatic disease characterized by overlapping manifestations of systemic lupus erythematosus (SLE), systemic sclerosis, and polymyositis/dermatomyositis, with high‐titer anti‐U1 ribonucleoprotein (U1‐RNP) antibodies [1, 2]. Several diagnostic and classification approaches have been proposed, including the Sharp, Kasukawa, Alarcón‐Segovia, and Japanese frameworks [3–5]. The revised 2019 Japanese criteria organize MCTD around common manifestations, anti‐U1‐RNP positivity, characteristic organ involvement, and overlapping SLE‐, systemic sclerosis‐, and polymyositis/dermatomyositis‐like features [5]. Trigeminal neuropathy is recognized as a characteristic neurological manifestation in the revised criteria [5]. Neurological involvement may include headache, seizures, encephalopathy, aseptic meningitis, peripheral neuropathy, and trigeminal neuropathy [6, 7]. Central nervous system (CNS) vasculitis is uncommon and has been reported only rarely [8–10]. Here, we describe a patient with longstanding systemic symptoms who developed acute neurological deterioration in the setting of probable MCTD‐associated CNS vasculitis.

## 2. Case Presentation

A 27‐year‐old woman, previously without a diagnosed disease, presented with small‐joint arthralgia of seven years’ duration. The symptoms were insidious in onset and associated with morning stiffness; she used analgesics intermittently for symptomatic relief. Arthralgia began in the right hand and, during the following year, progressed to involve the small joints of both hands and feet, accompanied by low‐grade fever and generalized weakness. Approximately three weeks before admission, she developed weakness of the left lower limb with limping and required assistance for walking. About two weeks before admission, she developed recurrent tonic–clonic seizures, inability to speak, fever, and altered sensorium. She was initially hospitalized elsewhere, where severe anemia was identified and treated with transfusion of two units of packed red blood cells. She subsequently developed weakness of the right upper and lower limbs and was referred to our hospital. There was no history of vomiting, headache, diplopia, or facial weakness.

There was no documented history of hypertension, diabetes mellitus, chronic kidney disease, or thyroid disease. Tuberculosis and known exposure to a person with tuberculosis were not reported. The available case record did not identify a prior rheumatological diagnosis or documented rheumatology evaluation during the past seven‐year history of symptoms. Before the current presentation, the only documented treatment for the chronic arthralgia was intermittent use of analgesics. No other long‐term immunomodulatory or disease‐specific rheumatological treatment was documented. Drug names and doses of the analgesics were not available in the case record.

On admission, the patient was drowsy. Blood pressure was 160/90 mmHg in both arms and 170/94 and 170/96 mmHg in the right and left legs, respectively. Pulse rate was 82/min, respiratory rate was 18/min, temperature was normal, and oxygen saturation was 96%. Cardiovascular and respiratory examinations were unremarkable. The Glasgow Coma Scale (GCS) score was E3M5V1. Pupils were equal, symmetrical, and reactive to light. No cranial nerve deficit or meningeal signs were elicited. She responded to painful stimuli in all four limbs but had right hemiparesis, with muscle strength of 2/5 in the right upper and lower limbs and 3/5 in the left limbs. Muscle tone was decreased bilaterally, and biceps, triceps, supinator, knee, and ankle reflexes were 1+ bilaterally. Bilateral plantar responses were mute. Sensory and cerebellar examinations could not be completed because of agitation.

Laboratory evaluation showed severe anemia (hemoglobin 6.8 g/dL), leukocytosis (14,700/µL), thrombocytopenia (119,000/µL), elevated erythrocyte sedimentation rate (48 mm/h) and quantitative C‐reactive protein (83 mg/L), elevated AST (79 U/L), alkaline phosphatase (268 U/L), and markedly elevated lactate dehydrogenase (1069 U/L). Serum calcium was low (7.09 mg/dL), triglycerides were elevated (338 mg/dL), and T3 was reduced (48 ng/dL). Renal function, HbA1c, and serum creatine phosphokinase were within the reported reference ranges. Direct and indirect Coombs tests were negative. The autoantibody profile showed positivity for nRNP‐Sm (2+) and AMA‐M2 (1+), while anti‐Sm, SS‐A, Ro‐52, SS‐B, Scl‐70, PM‐Scl100, Jo‐1, CENP‐B, PCNA, anti‐dsDNA, nucleosomes, histones, ribosomal P protein, p‐ANCA, and c‐ANCA were negative. Viral and other infectious disease screening, including HIV, anti‐HCV, HBsAg, malaria, dengue, typhoid, scrub typhus, and leptospirosis, was negative.

Urinalysis showed 2+ protein, 10–12 pus cells/high‐power field, and 20–25 red blood cells/high‐power field; urine culture was sterile. The reported 24‐h urinary protein was 1.1 gm. Chest radiography, two‐dimensional echocardiography, renal Doppler ultrasonography, and fundoscopy were normal. ECG showed sinus tachycardia. Abdominal ultrasonography demonstrated minimal ascites, bilateral minimal pleural effusions, mild hepatomegaly, and gallbladder sludge. Detailed results of other investigations are provided in Supporting Table S1.

Brain MRI demonstrated multiple confluent lesions involving the subcortical and periventricular white matter of both cerebral hemispheres. The lesions were hypointense on T1‐weighted images, hyperintense on T2‐weighted/FLAIR images, and showed restricted diffusion on DWI (Figure 1; Supporting Figure 1). We interpreted these findings in conjunction with the clinical, cerebrospinal fluid (CSF), and immunological data. The differential diagnosis included infectious and malignant/infiltrative CNS disease as potential mimics of an inflammatory or ischemic process. We evaluated infectious and malignant mimics using CSF CBNAAT, VDRL, cryptococcal antigen, malignant cytology, a pan‐neurotropic viral panel, and systemic infectious disease screening. However, the available record does not document a comprehensive systematic exclusion of every alternative cause of multifocal diffusion‐restricting lesions, including all embolic, hypertensive, demyelinating, or metabolic mechanisms; these alternatives therefore cannot be stated to have been definitively excluded.

**FIGURE 1:**
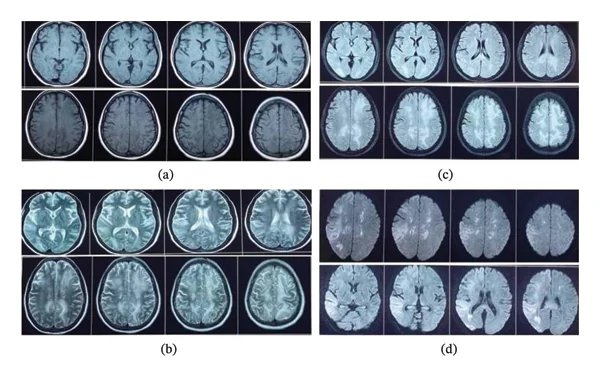
Multifocal (a) T1‐weighted hypointense, (b) T2‐weighted and (c) FLAIR hyperintense confluent lesions in the subcortical and periventricular white matter of both cerebral hemispheres; (d) lesions demonstrate restricted diffusion on DWI.

Treatment was initially administered empirically with broad‐spectrum antibiotics, antiseizure medication, antihypertensive therapy, and other supportive measures. After MCTD‐associated CNS vasculitis was considered probable on the basis of the clinical, serological, CSF, and neuroimaging findings, methylprednisolone pulse therapy was administered for five days, followed by oral prednisolone, and hydroxychloroquine was initiated. The patient showed marked clinical improvement, with a GCS score of E4M6V2 at discharge. Cyclophosphamide was planned as subsequent therapy; however, no follow‐up assessment was documented after discharge, and the available case record does not provide any information regarding its administration or subsequent clinical outcome. Accordingly, no neurological or systemic outcome beyond the documented discharge status is claimed.

## 3. Discussion

In this case, we report a 27‐year‐old woman with clinical and serological features consistent with MCTD who developed acute neurological deterioration characterized by seizures, altered sensorium, and hemiparesis. MCTD was considered on the basis of the patient’s overlapping systemic features and anti‐U1‐RNP antibody positivity, in the context of established diagnostic frameworks [3–5]. The 2019 Japanese criteria emphasize anti‐U1‐RNP positivity together with characteristic organ involvement and overlapping SLE‐, systemic sclerosis‐, and polymyositis/dermatomyositis‐like manifestations [5]. Trigeminal neuropathy is a characteristic neurological manifestation in these criteria [5]; however, trigeminal involvement was absent in the present case.

CNS involvement in MCTD may manifest with headache, encephalopathy, seizures, aseptic meningitis, focal neurological deficits, and peripheral neuropathy [6, 7]. CNS vasculitis is uncommon and has been described in association with MCTD and anti‐RNP autoimmunity [8–10]. MRI is an important noninvasive modality for demonstrating cerebral lesions, but MRI abnormalities are not specific for CNS vasculitis. Vascular imaging and, in selected cases, tissue biopsy may be required for stronger diagnostic confirmation [11, 12]. In the present patient, MCTD‐associated CNS vasculitis was considered probable from the combination of systemic autoimmune features, anti‐U1‐RNP positivity, inflammatory CSF abnormalities, negative infectious studies, and multifocal diffusion‐restricting MRI lesions. The patient’s laboratory evaluation demonstrated severe anemia, thrombocytopenia, markedly elevated inflammatory markers, and elevated LDH, while infectious investigations were negative. CSF showed lymphocyte‐predominant pleocytosis and substantially increased protein. Together with multifocal diffusion‐restricting white‐matter lesions, these findings supported an inflammatory/ischemic CNS process in the context of systemic autoimmunity. Proposed mechanisms of neurological involvement include immune‐mediated endothelial injury, inflammatory cytokine release, vascular inflammation, and disruption of cerebral perfusion and blood–brain barrier integrity; these mechanisms remain conceptual rather than proven in an individual patient [13, 14]. The broader clinical spectrum, diagnostic considerations, and therapeutic approaches to MCTD have been reviewed previously [15–17]. Long‐term outcome data in MCTD demonstrate that disease manifestations may evolve [18], while studies of anti‐RNP autoimmunity have explored mechanisms of tissue injury [19]. Previous reports have also described severe neurological involvement or probable systemic vasculitis in MCTD [20–22].

Management of CNS vasculitis associated with MCTD is not standardized because of its rarity [15–17]. In this patient, treatment included antiseizure and supportive therapy followed by high‐dose corticosteroids and hydroxychloroquine, with improvement in neurological status by discharge. Cyclophosphamide was planned; however, the available medical record contains no documented follow‐up after discharge or following its administration. Consequently, the longer‐term neurological or systemic response to therapy cannot be assessed. The principal limitation of this report is the lack of long‐term follow‐up, which limits assessment of the patient’s subsequent clinical course and outcome. Nevertheless, the case highlights the importance of integrating clinical, immunological, CSF (Table 1), and MRI findings to support a comprehensive diagnostic evaluation.

**TABLE 1:** Cerebrospinal fluid (CSF) profile of the patient.

No.	Parameter	Result
1	Total leukocyte count (TLC)	10 cells/mm^3^
2	Differential leukocyte count (DLC)	Neutrophils 5%; lymphocytes 95%
3	Glucose	56 mg/dL
4	Protein	259 mg/dL
5	CBNAAT	Not detected
6	VDRL	Negative
7	Malignant cytology	Negative
8	Cryptococcal antigen	Negative
9	Corresponding blood glucose	109 mg/dL
10	CSF opening pressure	18 cm H2O
11	CSF oligoclonal bands	Negative
12	Pan‐neurotropic viral panel	Negative

*Note:* CSF, cerebrospinal fluid.

Abbreviations: CBNAAT, cartridge‐based nucleic acid amplification test; DLC, differential leukocyte count; TLC, total leukocyte count; VDRL, Venereal Disease Research Laboratory test.

**Nomenclature**
ANA: Antinuclear antibodies
CSF: Cerebrospinal fluid
CNS: Central nervous system
MCTD: Mixed connective tissue disease
MRI: Magnetic resonance imaging
SLE: Systemic lupus erythematosus
U1‐RNP: U1 small nuclear ribonucleoprotein
AQP4: Aquaporin‐4
EMG: Electromyography
HLA: Human leukocyte antigen

## Author Contributions

CRediT authorship contribution statement: Arpan Mitra: conceptualization, investigation, and writing–original draft. Niraj Kumar Srivastava: conceptualization, investigation, and writing–original draft. Ankur Vivek: conceptualization, investigation, and writing–review and editing. Nayana Bhuyan: conceptualization, investigation, and writing–review and editing. Rukhsar Parveen: conceptualization, investigation, and writing–review and editing. Vijaya Nath Mishra: conceptualization, investigation, and writing–review and editing. Abhishek Pathak: conceptualization, supervision, and writing–review and editing.

## Funding

No funding was received for this manuscript.

## Disclosure

All authors have approved the final version of the manuscript.

## Consent

Written informed consent was obtained from the patient for publication of this case report and accompanying clinical details/images.

## Conflicts of Interest

The authors declare no conflicts of interest.

## Supporting Information

Additional supporting information can be found online in the Supporting Information section.

## Supporting information


**Supporting Information 1** Supporting Figure 1. Brain MRI findings in a patient with probable central nervous system vasculitis associated with mixed connective tissue disease (MCTD). Axial T2‐weighted and T1‐weighted images (A), FLAIR and diffusion‐weighted images (B, C), and post‐contrast T1‐weighted images (D) demonstrate multifocal cortical and subcortical signal abnormalities, predominantly involving the right cerebral hemisphere, with T2/FLAIR hyperintensity and corresponding areas of restricted diffusion, most prominent in the temporoparietal and posterior cerebral regions, consistent with acute/subacute ischemic injury. No definite focal mass lesion or abnormal parenchymal/leptomeningeal enhancement is evident on the provided postcontrast images. The imaging pattern, in the appropriate clinical context, is compatible with ischemic parenchymal injury associated with CNS vasculitis, although vascular imaging confirmation is not documented.

**Supporting Information 2** Supporting Table S1. Other investigations performed during the evaluation of the patient.

## Data Availability

The data that support the findings of this study are available from the corresponding author upon reasonable request.
