# Supplementary material for: Mixed Connective Tissue Disease (MCTD): A Rare Presentation of Probable Central Nervous System (CNS) Vasculitis
Source: Case Rep Neurol Med. 2026 Oct 17;2026:1782813. doi: 10.1155/crnm/1782813 (PMC13675906; doi:10.1155/crnm/1782813)
Supplement: Supplementary file 1 [file CRNM-2026-1782813-s002.docx]

**Supplementary Table S1. Other investigations performed during the evaluation of the patient**

| **No.** | **Investigation** | **Result** |
| --- | --- | --- |
| 1 | CXR AP view | Normal |
| 2 | USG abdomen | Minimal ascites, bilateral minimal pleural effusion, mild hepatomegaly with gallbladder sludge |
| 3 | ECG | Sinus tachycardia |
| 4 | 2D-ECHO | Normal |
| 5 | USG renal Doppler | Normal |
| 6 | Blood culture and sensitivity | Sterile |
| 7 | Fundoscopy | Normal |

**Abbreviations**: CXR, chest X-ray; AP, anteroposterior; USG, ultrasonography; ECG, electrocardiography; 2D-ECHO, two-dimensional echocardiography; C/S, culture and sensitivity.
